# A comparison of national essential medicines lists in the Americas

**DOI:** 10.26633/RPSP.2020.5

**Published:** 2020-01-27

**Authors:** Liane Steiner, Darshanand Maraj, Hannah Woods, Jordan Jarvis, Hannah Yaphe, Itunu Adekoya, Anjli Bali, Nav Persaud

**Affiliations:** 1 MAP Centre for Urban Health Solution St. Michael’s Hospital TorontoOntario Canada MAP Centre for Urban Health Solution, St. Michael’s Hospital, Toronto, Ontario, Canada.; 2 Department of Family and Community Medicine St. Michael’s Hospital and the University of Toronto TorontoOntario Canada Department of Family and Community Medicine, St. Michael’s Hospital and the University of Toronto, Toronto, Ontario, Canada.

**Keywords:** Formulary, Americas, access to essential medicines and health technologies, World Health Organization, Pan American Health Organization., Formulario farmacéutico, Américas, acceso a medicamentos esenciales y tecnologías sanitarias, Organización Mundial de la Salud, Organización Panamericana de la Salud, Formulário farmacêutico, Américas, acesso a medicamentos essenciais e tecnologias em saúde, Organização Mundial da Saúde, Organização Pan-Americana da Saúde

## Abstract

**Objectives.:**

To compare national essential medicines lists (NEMLs) from countries in the Region of the Americas and to identify potential opportunities for improving those lists.

**Methods.:**

In June of 2017, NEMLs from 31 countries in the Americas were abstracted from documents included in a World Health Organization (WHO) repository. The lists from the Americas were compared to each other and to NEMLs from outside of the Americas, as well as with the WHO Model List of Essential Medicines, 20^th^ edition (“WHO Model List”) and the list of the Pan American Health Organization (PAHO) Regional Revolving Fund for Strategic Public Health Supplies (“Strategic Fund”).

**Results.:**

The number of differences between the NEMLs from the Americas and the WHO Model List were similar within those countries (median: 295; interquartile range (IQR): 265 to 347). The NEMLs from the Americas were generally similar to each other. While the NEMLs from the Americas coincided well with the Strategic Fund list, some medicines were not included on any of those NEMLs. All the NEMLs in the Americas included some medicines that were withdrawn due to adverse effects by a national regulatory body (median: 8 withdrawn medicines per NEML; IQR: 4 to 12).

**Conclusions.:**

The NEMLs in the Americas were fairly similar to each other and to the WHO Model List and the Strategic Fund list. However, some areas of treatment and some specific medicines were identified that the countries should reassess when revising their NEMLs.

## RESUMEN

**Objetivos.:**

Comparar las listas nacionales de medicamentos esenciales (LNME) de países de la Regi delas Américas e identificar oportunidades potenciales de mejorarlas.

**Métodos.:**

En junio de 2017, se extrajeron las LNME de 31 países de la Regi de documentos incluidos en un repositorio de la Organizaci Mundial de la Salud (OMS). Se compararon estas listas entre sí y con listas de fuera de la Regi, así como con la Lista Modelo de Medicamentos Esenciales de la OMS (20^a^ edicion) y la lista del Fondo Rotatorio Regional para Suministros Estratégicos de Salud Plica de la Organizaci Panamericana de la Salud.

**Resultados.:**

El nero de diferencias entre las LNME de la Regi y la Lista Modelo de la OMS fue similar dentro de esos países (mediana: 295; rango intercuartil (RIC): 265 a 347). Las LNME de la Regi en general fueron similares entre sí. Si bien las LNME de la Regi mostraron una coincidencia adecuada con la lista del Fondo Rotatorio, algunos medicamentos no estaban incluidos en ninguna de las primeras. Todas las LNME de la Regi incluían algunos medicamentos que habían sido retirados del mercado por las autoridades regulatorias nacionales debido a efectos adversos (mediana: 8 medicamentos retirados en cada lista; RIC: 4 a 12).

**Conclusiones.:**

Las LNME en la Región de las Américas son bastante similares entre sí y con la Lista Modelo de la OMS y la lista del Fondo Rotatorio de la OPS. Sin embargo, se identificaron algunas áreas terapéuticas y algunos medicamentos específicos que los países deberían reevaluar al revisar sus LNME.

## RESUMO

**Objetivos.:**

Comparar as listas nacionais de medicamentos essenciais (LNME) dos países da Região das Américas e identificar oportunidades potenciais de melhoria.

**Métodos.:**

Em junho de 2017, as LNME de 31 países das Américas foram obtidas de documentos incluídos em um repositio da Organização Mundial da Sae (OMS). As listas foram comparadas entre si, com listas de fora da Região, com a Lista Modelo de Medicamentos Essenciais da OMS (20^a^ edição) e com a lista do Fundo Rotativo Regional para Fornecimentos Estratégicos de Sae Plica da Organização Pan-Americana da Sae (Fundo Estratégico).

**Resultados.:**

As LNME dos países das Américas eram semelhantes entre si e apresentaram um nero semelhante de diferenças em relação à Lista Modelo da OMS (mediana: 295; intervalo interquartil: 265-347). Embora as LNME nas Américas fossem altamente consistentes com a lista do Fundo Estratégico, alguns dos medicamentos do Fundo não apareciam em nenhuma dessas LNME. Todas as LNME nas Américas incluíam medicamentos retirados do mercado por algum organismo regulador nacional devido a efeitos adversos (mediana: 8 medicamentos retirados por LNME; intervalo interquartil: 4-12).

**Conclusões.:**

As LNME nas Américas são bastante semelhantes entre si e próximas da Lista Modelo da OMS e da lista do Fundo Estratégico. Contudo, foram identificadas algumas áreas terapêuticas e alguns medica mentos específicos que os países deveriam reavaliar ao rever as suas LNME.

Essential medicines lists are meant to promote equity in health by ensuring that quality medicines are available and accessible in a functioning health system, in appropriate forms, at affordable prices, and distributed in an equitable fashion (1). The World Health Organization (WHO) Model List of Essential Medicines (“WHO Model List”) (2) is revised biannually (3). The WHO Model List serves as a guide for countries’ national essential medicines lists (NEMLs), which prioritize a core set of medicines based on each country’s health needs (1, 4). NEMLs are used to guide medicine selection, appropriate use, medicine reimbursement, and medicine procurement, and they should be regularly updated (5, 6).

NEMLs guide medicine access for over 600 million people in the Region of the Americas (7). All countries with an NEML in the Americas are Member States of the Pan American Health Organization (PAHO), which works with countries to improve and protect the health of the people in their nation or territory (8).

In total, PAHO consists of 49 countries and territories (35 Member States plus 14 others categorized as participating, associate, or observer states) (9). PAHO promotes evidence-based choices for the countries’ NEML medicine selection through its Regional Revolving Fund for Strategic Public Health Supplies (the “Strategic Fund”) (10). The Strategic Fund has created its own list of medicines, based on the WHO Model List (11), that are available for procurement on behalf of PAHO Member States to leverage economies of scale in order to assist countries in the acquisition of quality, safe and effective medicines and other health supplies and services at affordable prices (12). The Strategic Fund also aims to build capacity at the national level for drug supply management and procurement programming and planning (12).

To our knowledge, no studies have compared NEMLs across countries in the Americas. This study sought to compare available NEMLs in the Americas with the WHO Model List (20^th^ edition, 2017) to determine potential recommendations for NEMLs in the Americas.

## METHODS

### Creation of the database

In June of 2017 we searched the WHO Essential Medicines and Health Products Information Portal, an online repository that contains hundreds of publications on medicines and health products related to WHO priorities (13, 14). We included all NEMLs that were posted on that repository, irrespective of their publication date and language. When more than one NEML was found for a country, the most recent was used. Detailed explanation of these methods is described elsewhere (15). The original database was updated with an NEML from Panama, and with other minor corrections.

### Exclusion criteria

We excluded documents that were not NEMLs, such as prescribing guidelines, and some medicines, including diagnostic agents, antiseptics, disinfectants, and saline solutions.

### Data extraction

From each country’s NEML, medicines were abstracted using International Nonproprietary Names (16). For medicines whose names were not in English we used the Anatomical Therapeutic Chemical (ATC) classification system, if available, or translated the names with the help of the Google Translate website (17, 18). Each medicine was listed individually, whether it was part of a combination product or not. Medicine bases and their salts were combined (e.g., promethazine hydrochloride and promethazine), as well as different compounds of the same vitamin or mineral (e.g., ferrous fumarate and ferrous sulfate). Detailed methods for the creation of the database, including data extraction, are described elsewhere (15).

### Data analysis

We determined the number of differences between each country’s NEML and the WHO Model List (20^th^ edition, 2017), including both the number of medicines on the WHO Model List but not on the respective NEML and the number of medicines on the respective NEML but not on the WHO Model List. Also for the countries in the Americas we determined the number of medicines that were on each NEML and on the PAHO Strategic Fund list, and the number of medicines that were not on each NEML but were on the Strategic Fund list.

Similarity scores were calculated for the 31 countries in the Americas by dividing medicines into two groups, those that were commonly listed (≥ 50%) and those that were uncommonly listed (< 50%). For each country’s NEML, a similarity score was calculated by totaling commonly listed medicines and uncommonly listed medicines, and then subtracting uncommonly listed from commonly listed medicines. Higher numbers indicate more similarity and lower scores indicate less similarity, in comparison to other NEMLs in the Americas.

Withdrawn medicines on NEMLs were identified in a previous study (19). We determined which ones were present on NEMLs in the Americas and calculated the number present on each country’s NEML.

## RESULTS

We included NEMLs from 138 countries: 31 in the Americas (89% of the 35 PAHO Member States) and 107 outside of the Americas (15). PAHO Member States that did not have an NEML in the online WHO NEMLs repository were the Bahamas, Canada, Guatemala, and the United States of America. The publication years for the NEMLs in the Americas ranged from 2004 to 2017.

The number of medicines on the individual lists from the Americas lists ranged from 197 to 704 (median: 361; interquartile range (IQR): 290 to 456) (Table 1). In comparison, the values for other world regions were: Africa, 64 to 702 (median: 298; IQR: 248 to 347); Eastern Mediterranean, 200 to 964 (median: 462; IQR: 278 to 623); Europe, 181 to 980 (median: 398; IQR: 285 to 601); Southeast Asia, 44 to 546 (median: 291; IQR: 230 to 343); and Western Pacific, 177 to 742 (median: 249; IQR: 215 to 295) (15).

In total, the 138 NEMLs contained 2 081 unique medicines. We identified 1 264 medicines included on the lists of the Americas, of which more than two-fifths (541; 43%) were listed by 3 or fewer countries.

### Comparison with the WHO Model List

We determined the number of differences between each country’s NEML and the WHO Model List, including both the number of medicines on the WHO Model List but not on the NEML and the number of medicines on the NEML but not on the WHO Model List (15). At the time of our study, the WHO Model List had 415 medicines on it. The number of differences between NEMLs in the Americas and the WHO Model List ranged from 175 to 531 (median: 295; IQR: 265 to 347) (Table 1). The values for the other world regions were: Africa, 208 to 538 (median: 281; IQR: 267 to 323); Eastern Mediterranean, 93 to 753 (median: 352; IQR: 249 to 502); Europe, 211 to 813 (median: 415; IQR: 337 to 535); Southeast Asia, 231 to 463 (median: 273; IQR: 243 to 329); and Western Pacific, 239 to 595 (median: 307; IQR: 284 to 347) (15).

**TABLE 1.** National essential medicines lists in the Region of the Americas

Country	ISO-3^a^ Country code	Health expenditure per capita ($Intl; 2015)^b^	NEML Year	Total number of medicines on NEML	Total differences from WHO Model List	No. of medicines on WHO Model List, but not on NEML	No. of medicines on NEML, but not on WHO Model List	No. of medicines on Strategic Fund list	No. of medicines on Strategic Fund list but not on NEML	Similarity score among countries
Antigua and Barbuda	ATG	1 105	2007	292	291	207	84	90	90	204
Argentina	ARG	1 390	2011	469	312	129	183	126	54	113
Barbados	BRB	1 234	2011	624	507	149	358	118	62	−60
Belize	BLZ	524	2008	370	279	162	117	110	70	134
Bolivia (Plurinational State of)	BOL	446	2011	353	270	166	104	124	56	169
Brazil	BRA	1 392	2014	406	347	178	169	117	63	12
Chile	CHL	1 903	2005	349	314	190	124	110	70	153
Colombia	COL	853	2011	371	288	166	122	120	60	125
Costa Rica	CRI	1 287	2014	389	354	190	164	108	72	85
Cuba	CUB	2 479	2012	505	358	134	224	133	47	45
Dominica	DMA	586	2007	284	295	213	82	89	91	202
Dominican Republic	DOM	873	2015	356	175	117	58	136	44	172
Ecuador	ECU	980	2013	370	243	144	99	133	47	118
El Salvador	SLV	579	2009	361	268	161	107	116	64	157
Grenada	GRD	678	2007	282	303	218	85	85	95	196
Guyana	GUY	336	2010	280	265	200	65	96	84	156
Haiti	HTI	120	2012	197	248	233	15	88	92	155
Honduras	HND	353	2009	366	325	187	138	106	74	112
Jamaica	JAM	511	2012	456	343	151	192	122	58	104
Mexico	MEX	1 009	2011	704	531	121	410	133	47	−162
Nicaragua	NIC	406	2011	272	261	202	59	106	74	180
Panama	PAN	1 543	2017	601	398	106	292	143	37	−65
Paraguay	PRY	724	2009	307	272	190	82	114	66	161
Peru	PER	671	2012	424	243	117	126	140	40	138
Saint Kitts and Nevis	KNA	1 443	2007	290	297	211	86	89	91	206
Saint Lucia	LCA	681	2007	290	297	211	86	89	91	206
Saint Vincent and the Grenadines	VCT	470	2010	267	250	199	51	100	80	201
Suriname	SUR	1 017	2014	285	260	195	65	103	77	151
Trinidad and Tobago	TTO	2 204	2010	492	377	150	227	122	58	58
Uruguay	URY	1 748	2011	518	445	171	274	109	71	−14
Venezuela (Bolivarian Republic of)	VEN	579	2004	306	289	199	90	116	64	160

***Table 1 notes:*** ISO-3: International Organization for Standardization alpha-3 letter codes; $Intl: International dollars; NEML: national essential medicines list; Strategic Fund List (refers to the Pan American Health Organization - Regional Revolving Fund for Strategic Public Health Supplies); WHO: World Health Organization.

***Source:*** Authors’ results and data from publicly available sources as summarized below:

a We obtained the countries’ alpha three-letter codes from the International Organization for Standardization (ISO) 3166-1, Online Browsing Platform.

b Health care expenditure per capital from the WHO/Global Health Observatory data repository.

In the Americas, 23 medicines from the WHO Model List were not included on any NEML (Table 2) (15). The Dominican Republic, Mexico, and Peru included more than 290 (70%) of the medicines on the WHO Model List on their NEMLs. Of these NEMLs, the Dominican Republic and Peru listed medicines from the WHO Model List without adding many other medicines (fewer than 130), while Mexico added over 400 medicines to its list that were not on the WHO Model List. Dominica, Grenada, Haiti, Saint Kitts and Nevis, and Saint Lucia omitted over 207 (50%) of the medicines on the WHO Model List from their NEMLs (Table 1).

For neglected tropical diseases, there were two medicines (benznidazole and nifurtimox) for American trypanosomiasis (Chagas disease) and five antileishmaniasis medicines (amphotericin B, miltefosine, paromomycin, meglumine, and stibogluconate) on the WHO Model List. Ten countries in the Americas listed at least one medicine to treat Chagas disease, while 30 countries listed at least one medicine to treat leishmaniasis. Haiti was the only country that did not list any treatment for either disease.

**FIGURE 1.**
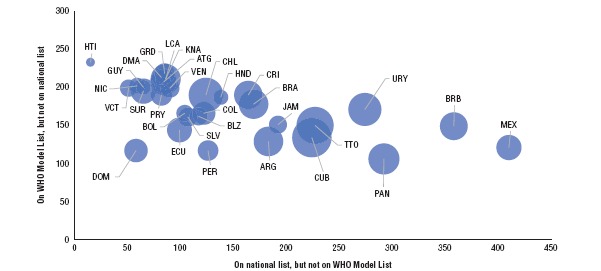
Health expenditure and dissimilarities between national essential medicines lists of the Americas and WHO Model List

Countries in the Americas with lower health care expenditures appear to have omitted more WHO essential medicines from their lists (e.g., Haiti and Nicaragua), and countries with higher health care expenditures appear to have included more medicines on their lists that are not on the WHO Model List (e.g., Barbados, Mexico, Panama, and Uruguay), although there are exceptions (e.g., Antigua and Barbuda) (Figure 1).

### Comparison with the Strategic Fund list

The Strategic Fund list includes 180 unique medicines; 95% of listed medicines are also included on the WHO Model List. The number of medicines listed on both the Strategic Fund list included on any national essential medicines list in the Region and a specific country’s NEML ranged from 85 to 143 (median: of the Americas 114; IQR: 100 to 124) (Table 1). Six countries (Cuba, Dominican Republic, Ecuador, Mexico, Panama, and Peru) included more than 126 (70%) of the Strategic Fund medicines on their respective NEML.

**TABLE 2.** Medicines listed by the WHO Model List but not included on any national essential medicines list in the Region of the Americas

Medicines (ATC code)	Strategic Fund listed
Artenimol/Dihydroartemisinin (P01BE05)	Yes
Bedaquiline (J04AK05)	Yes
Delamanid (J04AK06)	Yes
Dolutegravir (J05AX12)	Yes
Mifepristone (G03XB01)	Yes
Piperaquine (P01BX02)	Yes
Protionamide/Prothionamide (J04AD01)	Yes
Rifapentine (J04AB05)	Yes
Velpatasvir (J05AP55)	Yes
Ceftaroline (J01DI02)	No
Cyclizine (R06AE03)	No
Daptomycin (J01XX09)	No
Eflornithine (P01CX03)	No
Etonogestrel-releasing implant (G03AC08)	No
Faropenem (J01DI03)	No
Japanese encephalitis vaccine (J07BA02)	No
Melarsoprol (P01CD01)	No
Progesterone vaginal ring (G03DA04)	No
Protease (A09AA02)	No
Pyronaridine (P01BF06)	No
Suramin (P01CX02)	No
Tick-borne encephalitis immunoglobulin (J06BB12)	No
Ulipristal (G03AD02)	No

ATC: Anatomical Therapeutic Chemical classification system. WHO Model List refers to the World Health Organization’s Model List of Essential Medicines, 20th version (2017); Strategic Fund listed indicates that the medicines are included on the Pan American Health Organization (PAHO) Strategic Fund Medicine List (September 2018). ***Source:*** Authors’ results.

The number of medicines listed on the Strategic Fund list and not on a specific country’s NEML ranged from 37 to 95 (median: 66; IQR: 56 to 80) (Table 1). We identified 9 medicines that are listed on both the Strategic Fund list and the WHO Model List but not by any country in the Americas (Table 2); these medicines are commonly used in the treatment of hepatitis C, tuberculosis, human immunodeficiency virus, and malaria. Atovaquone (ATC: P01AX06), which is used in the treatment of malaria, pneumocystis pneumonia, and toxoplasmosis, is not on the WHO Model List or any NEML in the Americas but is included on the Strategic Fund list.

Velpatasvir, a medicine used in combination for treatment of hepatitis C, was not listed by any NEML in the Americas. Seven other medicines for hepatitis C (daclatasvir, dasabuvir, ledipasvir, ombitasvir, paritaprevir, sofosbuvir, and simeprevir) were each listed by fewer than three countries. All of these medicines for hepatitis C treatment are included on the Strategic Fund list. The other medicines included on the WHO Model List for the treatment of hepatitis C are ribavirin and pegylated interferon alfa, which are used in combination; only six countries in the Americas (Brazil, Cuba, Honduras, Mexico, Panama, and Uruguay) listed these medicines.

WHO: World Health Organization (Model List of Essential Medicines, 20th edition, 2017).

The width of the circles represents the country’s health expenditure. We obtained the countries’ three-letter codes from the International Organization for Standardization (ISO) 3166-1 Online Browsing Platform; ATG: Antigua and Barbuda; ARG: Argentina; BRB: Barbados; BLZ: Belize; BOL: Bolivia (Plurinational State of); BRA: Brazil; CHL: Chile; COL: Colombia; CRI: Costa Rica; CUB: Cuba; DMA: Dominica; DOM: Dominican Republic; ECU: Ecuador; SLV: El Salvador; GRD: Grenada; GUY: Guyana; HTI: Haiti; HND: Honduras; JAM: Jamaica; MEX: Mexico; NIC: Nicaragua; PAN: Panama; PER: Peru; PRY: Paraguay; KNA: Saint Kitts and Nevis; LCA: Saint Lucia; VCT: Saint Vincent and the Grenadines; SUR: Suriname; TTO: Trinidad and Tobago; URY: Uruguay; VEN: Venezuela (Bolivarian Republic of).

***Source:*** Authors’ results.

Four antituberculosis medicines rifapentine (treatment of protionamide (part of treatment regimens for multidrug and extensively drug-resistant tuberculosis)) are not listed on any NEML in the Americas. These medicines are included on the WHO Model List and the Strategic Fund list.

### Between-country comparisons

The similarity scores for the Americas, measuring the extent to which countries tend to list medicines commonly listed by other countries in the Americas, ranged from −162 to 206 (median: 151; IQR: 85 to 172) (Table 1). Most countries in the Americas had a positive similarity score, indicating that most of the medicines listed by those countries were also listed by the majority of countries included in this analysis. Mexico had a large negative similarity score (−162), indicating that the majority of medicines on its list were not listed by most countries in the Americas.

### Discrepant medicines

We identified medicines that could be added or removed from NEMLs in the Americas by calculating whether medicines were commonly listed (listed by ≥ 50% of countries) in each WHO region. Medicines that are not commonly listed in the Americas but are on the WHO Model List and commonly listed by at least three other regions (50% of the WHO regions) could be considered for addition (Table 3). Medicines commonly listed within the Americas but not on the WHO Model List or not commonly listed by any other region could be considered for removal (Table 3).

Withdrawn medicines are those that have been withdrawn after market approval or those that were not approved by a national regulatory body because of adverse effects (19). Withdrawn medicines were present on all the NEMLs of the Americas; these ranged from 2 to 24 medicines (median: 8; IQR: 4 to 12) (Table 4). Of the 31 studied countries in the Americas, 10 of them (32%) listed 11 or more withdrawn medicines. Three internationally withdrawn medications were present on NEMLs in the Americas: drotrecogin alfa (Mexico); nikethamide (Cuba); and thioridazine, an antipsychotic that was withdrawn by the manufacturer (Novartis) in 2005 (19), (listed by 18 of the 31 countries in the Americas (58%)).

## DISCUSSION

We found that NEML listings across countries in the Americas are similar to each other, with a few exceptions (including Barbados, Mexico, Panama, and Uruguay), and similar to the WHO Model List (Table 1). Some medicines listed by multiple countries in the Americas could be considered for removal because they are not listed in either the WHO Model List or by many other countries (e.g., trifluoperazine) or because they have been withdrawn in other countries (e.g., thioridazine). Some medicines that are not listed by many countries in the Americas should be considered for addition, such as medicines on the Strategic Fund list (e.g., tuberculosis treatments).

The WHO states that medicine availability and price are key indicators of access to treatment (20). NEMLs are commonly used to guide public sector procurement (20), and they have been shown to be more available than other medicines, particularly in the public sector (21). Therefore, the content of an NEML can affect health outcomes.

**TABLE 3.** Medicines that may be considered for addition or removal from national essential medicines lists in the Region of the Americas, with the medicines’ Anatomical Therapeutic Chemical classification system code

Possible action/Medicines
Potential additions
Calamine (D04AX)
Cefixime (J01DD08)
Clomifene/Clomiphene (G03GB02)
Clomipramine (N06AA04)
Copper IUD (G02BA02)
Cycloserine (J04AB01)
Ephedrine (C01CA26)
Niclosamide (P02DA01)
Ofloxacin (J01MA01)
Rabies vaccine (J07BG)
Tetracaine (C05AD02)
Vecuronium (M03AC03)
Potential removals
Amino acids (B05BA01)
Dimenhydramine/Dimenhydrinate (R06AA52)
Diphenhydramine (R06AA02)
Glutaraldehyde (D08AX09)
Indinavir (J05AE02)
Labetalol (C07AG01)
Metamizole/Dipyrone (N02BB02)
Methylphenidate (N06BA04)
Mitomycin (L01DC03)
Nelfinavir (J05AE04)
Proxymetacaine/Proparacaine (S01HA04)
Sevoflurane (N01AB08)
Thalidomide (L04AX02)
Thioridazine (N05AC02)
Trifluoperazine (N05AB06)

***Source:*** Authors’ results.

The WHO recommends that their Model List be used to guide countries to the best evidence-based medicines and not necessarily be replicated on NEMLs. Countries should select medicines based on their own, specific priority health care needs (20). Having a larger overlap of medicines with the WHO Model List may not guarantee better health outcomes for countries. Health care services and quality of care are also important factors that may affect health outcomes. The purpose of a comparison with the WHO Model List is to encourage countries to evaluate their list based on our findings to ensure that their NEMLs use the best evidence-based medicines to meet the needs of their country. We acknowledge that there are other factors, such as relationships with pharmaceutical manufacturers and structural differences within health systems, that may influence NEML listing decisions.

### List comparisons

Within the Americas, Mexico’s NEML was the least like those of other countries, as it was the longest (at 704 medicines) and it had the lowest similarity score. Countries may find that with a longer NEML it is harder to maintain an adequate and consistent supply of medicines, therefore the WHO recommends that countries list a limited number of carefully selected medicines (20). The reasons for Mexico’s longer list are not clear; Mexico is not the wealthiest country in the Americas, nor does it have the largest population (7). Policy process or political factors, such as limited use of evidence-based processes in selecting medicines, as has been demonstrated in other settings, may explain observed differences among NEMLs (22).

**TABLE 4.** Withdrawn medicines included on national essential medicines lists in the Region of the Americas

Withdrawn medicine (ATC code)	Safety concern	Countries listing the medicine (no.)
Benzbromarone (M04AB03)	Hepatic damage	Panama (1)
Benzbromarone (M04AB03)	Hepatic damage	Panama (1)
Bismuth (A02BX05)	Encephalopathy	Argentina, Belize, Cuba, Jamaica, Mexico, Panama, Peru, Suriname, Trinidad and Tobago, Uruguay (10)
Carisoprodol (M03BA02)	Abuse potential	Guyana (1)
Chloral hydrate (N05CC01)	Tumorigenicity	Barbados, Belize, Chile, Costa Rica, Cuba, Ecuador, Honduras, Jamaica, Mexico, Panama (10)
Chlormadinone (G03DB06)	Tumorigenicity	Mexico (1)
Chloroform (N01AB02)	Cardiotoxicity, tumorigenicity	Trinidad and Tobago (1)
Cisapride (A03FA02)	Cardiac arrhythmias	Barbados, Mexico, Uruguay (3)
Clioquinol (D08AH30)	Subacute myelo-optic neuropathy (SMON), neurotoxicity	Barbados, Mexico, Panama, Trinidad and Tobago (4)
Clobutinol (R05DB03)	Long QT syndrome, cardiac arrhythmias	Chile (1)
Clofibrate (C10AB01)	Death, ventricular arrhythmias	Barbados, Uruguay (2)
Diclofenac (M01AB05)	Gastrointestinal, skin reactions	Antigua and Barbuda, Argentina, Barbados, Belize, Bolivia, Chile, Colombia, Costa Rica, Cuba, Dominica, Dominican Republic, Ecuador, El Salvador, Grenada, Guyana, Haiti, Honduras, Jamaica, Mexico, Nicaragua, Panama, Paraguay, Peru, Saint Kitts and Nevis, Saint Lucia, Saint Vincent and the Grenadines, Suriname, Trinidad and Tobago, Uruguay, Venezuela (Bolivarian Republic of) (30)
Dienestrol (G03CB01)	Carcinogenicity	Costa Rica (1)
Diethylstilbestrol/Stilboestrol (G03CB02)	Tumorigenicity	Argentina, Belize, Costa Rica, Cuba, Guyana, Jamaica, Panama, Peru (8)
Droperidol/Dehydrobenoperidol (N05AD08)	Cardiotoxicity	Argentina, Bolivia, Chile, Costa Rica, Cuba, Jamaica, Nicaragua (7)
Drotrecogin alfa (B01AD10)^*^	Failure to show benefits	Mexico (1)
Etretinate (D05BB01)	Teratogenicity	Trinidad and Tobago (1)
Fluvoxamine (N06AB08)	Teratogenicity, nephrotoxicity	Barbados, Panama, Trinidad and Tobago (3)
Furazolidone (G01AX06)	Carcinogenic, skin	Argentina, Colombia, Nicaragua, Peru (4)
Gatifloxacin (J01MA16)	Dysglycemia	Barbados (1)
Gemfibrozil (C10AB04)	Adverse effects not balanced by benefits	Argentina, Barbados, Belize, Bolivia, Brazil, Chile, Colombia, Costa Rica, Ecuador, Nicaragua, Paraguay, Peru, Venezuela (Bolivarian Republic of) (13)
Kaolin (A07BC02)	No evidence it works for its purpose	Trinidad and Tobago (1)
Ketorolac (M01AB15)	Gastrointestinal, skin reactions	Barbados, Bolivia, Dominican Republic, Ecuador, El Salvador, Honduras, Mexico, Nicaragua, Paraguay, Trinidad and Tobago (10)
Lindane/Gamma benzene hexachloride	Potential toxicity (P03AB02)	Barbados, Belize, Guyana, Jamaica, Uruguay, Venezuela (Bolivarian Republic of) (6)
Meclizine (R06AE05)	Teratogenic potential	Cuba (1)
Megestrol (G03AC05)	Tumorigenicity	Argentina, Barbados, Honduras, Mexico, Panama, Uruguay (6)
Meprobamate (N05BC01)	Abuse	Cuba (1)
Metamizole/Dipyrone (N02BB02)	Agranulocytosis	Belize, Bolivia, Brazil, Chile, Colombia, Costa Rica, Cuba, Guyana, Honduras, Jamaica, Mexico, Nicaragua, Panama, Paraguay, Peru, Uruguay, Venezuela (Bolivarian Republic of) (17)
Metaproterenol/Orciprenaline (R03AB03)	Cardiotoxicity	Mexico (1)
Methylphenidate (N06BA04)	Abuse	Antigua and Barbuda, Argentina, Barbados, Belize, Bolivia, Chile, Colombia, Costa Rica, Cuba, Dominica, El Salvador, Grenada, Honduras, Jamaica, Mexico, Panama, Peru, Saint Kitts and Nevis, Saint Lucia, Suriname, Trinidad and Tobago, Uruguay, Venezuela (Bolivarian Republic of) (23)
Minocycline (A01AB23)	Dizziness, vertigo	Argentina, Barbados, Brazil, Cuba, Mexico (5)
Neomycin (A01AB08)	Abuse	Antigua and Barbuda, Argentina, Barbados, Belize, Bolivia, Chile, Colombia, Costa Rica, Cuba, Dominica, El Salvador, Grenada, Guyana, Jamaica, Mexico, Saint Kitts and Nevis, Saint Lucia, Suriname, Trinidad and Tobago, Uruguay (20)
Nikethamide (R07AB02)^*^	Neurotoxicity	Cuba (1)
Nimesulide (M01AX17)	Hepatotoxicity	Venezuela (Bolivarian Republic of) (1)
Phenazopyridine (G04BX06)	Carcinogenicity	Barbados, Costa Rica, El Salvador, Jamaica, Mexico, Uruguay (6)
Phentolamine (C04AB01)	Carcinogenicity	Argentina, Colombia, Cuba, Trinidad and Tobago, Venezuela (Bolivarian Republic of) (5)
Phenylpropanolamine (R01BA01)	Hemorrhagic stroke	Mexico (1)
Phthalylsulfathiazole (A07AB02)	Granulocytopenia	Uruguay (1)
Pioglitazone (A10BG03)	Risk of bladder cancer	Barbados, Jamaica, Mexico, Uruguay (4)
Pseudoephedrine (R01BA02)	Neurotoxicity, gastrointestinal	Barbados, El Salvador, Jamaica (3)
Rimonabant (A08AX01)	Psychiatric	Mexico (1)
Rosiglitazone (A10BG02)	Cardiotoxicity	Barbados, Honduras, Mexico, Paraguay, Trinidad and Tobago (5)
Sulfacetamide (S01AB04)	Eye, skin reactions	Colombia, Cuba, Haiti, Jamaica, Mexico, Nicaragua, Peru (7)
Sulfathiazole (J01EB07)	Nephrotoxicity, hepatotoxicity, skin reactions	Cuba (1)
Tegaserod maleate (A06AX06)	Increased risk of heart attacks and strokes	Mexico (1)
Thalidomide (L04AX02)	Teratogenicity	Argentina, Barbados, Bolivia, Brazil, Colombia, Costa Rica, Cuba, Ecuador, El Salvador, Honduras, Mexico, Nicaragua, Panama, Paraguay, Peru, Suriname, Uruguay (17)
Thioridazine (N05AC02)^*^	Cardiac arrhythmias, QT prolongation	Antigua and Barbuda, Barbados, Belize, Bolivia, Chile, Colombia, Cuba, Dominica, Grenada, Guyana, Nicaragua, Peru, Saint Kitts and Nevis, Saint Lucia, Saint Vincent and the Grenadines, Trinidad and Tobago, Uruguay, Venezuela (Bolivarian Republic of) (18)
Tolcapone (N04BX01)	Hepatotoxicity	Brazil (1)
Tranylcypromine (N06AF04)	Drug-drug interactions	Argentina (1)
Trazodone (N06AX05)	Carcinogenicity	Colombia (1)
Triazolam (N05CD05)	Psychiatric adverse effects	Mexico (1)
Valdecoxib (M01AH03)	Cardiotoxicity, skin reactions	Panama (1)
Vigabatrin (N03AG04)	Neurotoxicity	Brazil, Costa Rica, Cuba, Mexico (4)
Zopiclone (N05CF01)	Carcinogenicity	Barbados (1)

ATC: Anatomical Therapeutic Chemical classification system.

* Medicine has been withdrawn worldwide.

***Source:*** Data retrieved from Charles et al. (19); data available from the corresponding author of this study.

There is fair overlap between medicines on the NEMLs and those with a negotiated price through the Strategic Fund list. This suggests that the Strategic Fund list may be influential in its intended goal of helping countries to improve access to some medicines. At the same time, some high-priced medicines on the Strategic Fund list are not on most of the NEMLs, including treatments for hepatitis C, human immunodeficiency virus, tuberculosis, and malaria. This suggests that further work may be needed to make these medicines affordable, and perhaps price concerns have held up their listings on NEMLs.

We found that hepatitis C medicine listings on NEMLs are lacking in the Americas, with only 5 of 31 countries in the Americas (16%) having at least one treatment for it. That is despite the fact that the Strategic Fund list includes medicines for treating hepatitis C. Due to medical advances, 95% of people infected with hepatitis C could be cured. However, across the Americas, the vast majority of infected people do not have affordable access to these highly effective medicines (23).

Mifepristone, which is used in emergency contraception and in therapeutic abortions, is included on the Strategic Fund list and on the WHO Model List (since 2006) with the note “where permitted under national law and where culturally acceptable”; it is not included by any country in the Americas.

Antituberculosis medicines like protionamide have been listed on the WHO Model List for decades. In addition, in April 2015, rifapentine (for latent tuberculosis infections) and bedaquiline and delamanid (latest second-line treatment for multidrug-resistant infection) were added to the WHO Model List (24); as such, only NEMLs published after 2015 may have included these medicines.

The Dominican Republic and Panama were the only countries in the Americas that had an NEML published in 2015 or later, as of the time that we captured our study data. Ultimately, the choice of medicines included on NEMLs resides with national policymakers, and it is the responsibility of countries to regularly update and publish their NEMLs.

Along with the medications identified for addition, when comparing NEMLs to the WHO Model List and the Strategic Fund list, other medicines were identified that countries could consider adding to their NEMLs (Table 3). If many regions outside of the Americas are commonly listing particular medicines (e.g., cefixime and ephedrine), there is a consensus that they are essential among those countries. Therefore, medicines that were commonly listed on NEMLs in other regions could be considered for addition to NEMLs in the Americas, keeping in mind each country’s epidemiological needs.

### NEML medicine removals

We assessed medicines that countries could consider removing from their NEMLs (Table 3). If many countries outside of the Americas are not commonly listing particular medicines (e.g., labetalol), there may be a consensus among those countries that they are not essential. Several medicines on NEMLs in the Americas were identified as not approved or as withdrawn from the market due to adverse effects of the medicine (e.g., chloral hydrate and thioridazine) (Table 4). NEMLs are meant to guide medicine prescribing (5, 6), and it is important that they be reviewed regularly and that medicines with questionable evidence be carefully considered for omission from the lists. In this way, known harms to the population that a list serves can be prevented.

Treatments for priority noncommunicable disease interventions identified in three WHO guidelines—Best Buys (25), PEN (26), and HEARTs technical package (5)—were present in most countries in the Americas, according to previous studies. Areas identified for improvement were influenza vaccination, human papillomavirus vaccine, and senna (sennosides) (27).

### Strengths and limitations

This is the first and largest study comparing the medicines included in 31 NEMLs in the Region of the Americas. The size of the study offers some robustness to the findings, particularly our suggestions for NEML revisions in the Americas.

Our study has limitations. The database of NEMLs and medicines may not reflect current NEML listings, given that documents available from the WHO’s NEML repository were abstracted in 2017. In addition, some NEMLs required translation, standardized medicine nomenclature was not consistently used on some lists, and judgments had to be made about what to include in ambiguous cases. These issues made the process liable to errors. This quantitative analysis does not account for other contextual factors that could explain differences in which medicines are included on each NEML, such as local disease prevalence or national priorities.

### Conclusions

Countries in the Americas have NEMLs that are similar and that have significant overlap with both the WHO and Strategic Fund lists. However, countries in the Americas were lacking NEML coverage of medicines for treatment of hepatitis C, human immunodeficiency virus, tuberculosis, and malaria. Regularly updating NEMLs (as recommended by the WHO) and purchasing medicines through the Strategic Fund may help improve access to essential medicines and universal health coverage in the Americas. This may lead to improvements in measurable health outcomes and ultimately better the health of people in the Americas.

### Recommendations

When updating their NEMLs, studied countries in the Americas should consider the differences between their respective NEML and the WHO Model List and the other NEMLs in the Americas. Countries should also assess adding or removing medications from their NEML based on listings in other WHO regions, and they should also weigh removing medications that were withdrawn, particularly ones that have been withdrawn worldwide.

### Author contributions.

NP, JJ, and DM conceived the original idea. LS, DM, HY, and IA collected and analyzed the data. All authors contributed to writing and reviewing the paper and approved the final version. We also acknowledge that the results have not been fully or partially published or submitted to any other printed or electronic publication, in any language.

### Financial support.

This research was funded by the Canadian Institutes of Health Research and Ontario SPOR (Strategy for Patient Oriented Research) Support Unit. The funder did not influence the design, data collection, analysis, writing, or decision to publish these results.

### Disclaimer.

Authors hold sole responsibility for the views expressed in the manuscript, which may not necessarily reflect the opinion or policy of Canadian Institutes of Health Research and Ontario SPOR Support Unit or the *RPSP/PAJPH* and/or PAHO.
